# Unusually large paraganglioma complicated with successive catecholamine crises: A case report and review of the literature

**DOI:** 10.3389/fsurg.2022.922112

**Published:** 2022-08-31

**Authors:** Zhenhui Huang, Guojian Liang, Hua Shen, Chuyuan Hong, Xuexia Yin, Shi Zhang

**Affiliations:** Department of Gastrointestinal Surgery, The Second Affiliated Hospital of Guangzhou Medical University, Guangzhou, China

**Keywords:** paraganglioma, extra-adrenal pheochromocytoma, catecholamine crisis, acute heart failure, neuroendocrine tumor, retro-peritoneal tumor

## Abstract

**Background:**

Paragangliomas are rare neuroendocrine tumors that could secret catecholamines. Hypertension and heart failure caused by the catecholamine crisis are fatal cardiovascular events. However, silent paragangliomas that lack typical symptoms of catecholamine pose a significant diagnostic challenge.

**Case summary:**

A 45-year-old woman who presented with more than 1-year history of abdominal discomfort was suspected of having a gastrointestinal stromal tumor by a local hospital since a vast metastatic mass occupied her left abdomen. Thus, she was recommended to our hospital. After completing the gastroscopy, she unexpectedly developed acute heart failure and was transferred to the Intensive Care Unit (ICU) where the initial diagnosis of paraganglioma was considered through path. However, a second catecholamine crisis due to constipation led to acute heart failure again. After anti-heart failure therapy and rigorous preoperative preparation, surgery was arranged to remove the tumor. Postoperative pathology conﬁrmed the paraganglioma, and the patient was discharged from the hospital in good condition.

**Conclusion:**

We reported a rare case of huge retro-peritoneal paraganglioma with successive catecholamine crises and acute heart failure. This was probably the largest retro-peritoneal paraganglioma since the 1980s. Besides, we were the first to use surgical drawing to illustrate its complex anatomical adjacent relationship of retro-peritoneal paraganglioma. Our case emphasizes the inclusion of extra-adrenal paraganglioma in the differential diagnosis of retroperitoneal tumors. In suspected paragangliomas, catecholamine testing is preferable to invasive procedures including gastroscopy and biopsy to avoid triggering a catecholamine crisis. Surgical resection is the primary treatment. We highlight the priority of dealing with the venous reflux branches of the tumor to prevent the release of catecholamines into the blood. In particular, preoperative preparation plays a vital role in managing paraganglioma. Moreover, it is necessary to schedule genetic testing and clinical follow-up due to the metastatic potential of paragangliomas.

## Introduction

Paragangliomas are rare neuroendocrine tumors arising from chromafﬁn cells of the parasympathetic or sympathetic paraganglia outside of the adrenal glands (1). Clinical symptoms include hypertension, tachycardia, headache, sweating, etc. The intermittent nature of these symptoms often leads to a delay in the diagnosis (2). Paraganglioma can cause catecholamines cardiomyopathy including myocardial infarction, heart failure, and arrhythmia (2). Catecholamine crisis is an uncommon and dreaded complication.

Herein, we described a rare and severe case of huge paraganglioma with two times catecholamine crises to underline the diagnosis and treatment of silent paragangliomas that lack typical manifestations.

## Case presentation

A 45-year-old woman was admitted to our hospital in February 2021, who presented with acid reflux in the past one month followed by more than a one-year history of abdominal discomfort after eating. She had normal menstruation without a history of hypertension, coronary heart disease, diabetes, or other diseases. There was no family history of illness. Suspected to be a gastrointestinal stromal tumor by the local hospital due to a vast mass occupying her abdomen through abdominal computed tomography (CT), she was recommended to our hospital for further diagnosis and treatment.

Vital signs on presentation were blood pressure=120/60 mmHg, heart rate=82 beats/min, and respiratory rate=20 times/min. On examination, the left abdominal mass was palpable with poor activity and unclear border. In addition, there is no tenderness and rebound pain in the abdomen. Moreover, all laboratory tests including blood routine, liver function, coagulation function, biochemistry even tumor markers did not show significant abnormalities.

However, after finishing the gastroscopy, she felt sudden pain and discomfort under the xiphoid accompanied by sweating, chest tightness, and shortness of breath that night. On examination, her heart rate was 110–127 beats/min, and respiratory rate was 20–24 times/min, and her blood pressure fluctuated from 117–179/78–91 mmHg (the maximum blood pressure was 179/91 mmHg), with blood oxygen was 90%–100% and her both lungs had no obvious rales on auscultation.

A series of emergent measures were taken immediately. Subsequently, laboratory tests revealed a significant increase in biomedical markers of myocardial injury, including type B natriuretic peptide pro-N-terminal (6980.00 ng/L, normal range 0–125 ng/L), cardiac troponin (15.60 μg/L, normal range 0–0.03 μg/L), myoglobin(291.7 μg/L, normal range 0–106 μg/L), alanine transaminase (177.00 U/L, normal range 13–14 U/L), lactate dehydrogenase (1015.00 U/L, normal range120–246 U/L), creatine kinase (933.20 U/L, normal range 40–200 U/L), and creatine kinase isoenzyme (147.70 U/L, normal range 0–25 U/L). The inﬂammatory markers were much higher than normal, such as the level of white blood cell count [22.62 × 109/L, normal range (4–10) × 109/L], and neutrophil ratio (90.60%, normal range 40%–75%). Then, we asked the cardiologist for urgent consultation, and he considered differential diagnoses, including acute myocardial infarction and acute heart failure. Due to the rapid heart rate and the progressive decline in blood oxygen, the patient was transferred to ICU for further monitoring and treatment.

**Figure 1:**
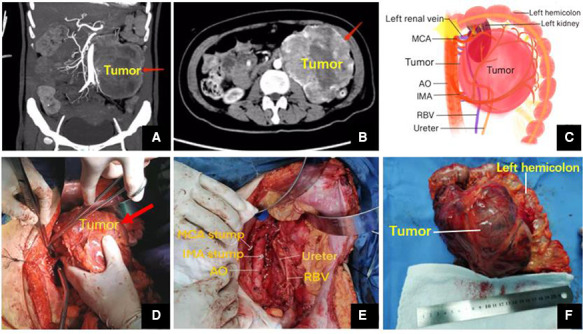
(**A,B**) Abdominal coronal and horizontal CT. (**C**) Postoperative surgical drawing. (**D,E**) Operative photos. (**F**) Gross examination revealed an encapsulated elliptic mass (17.0 cm × 13.1 cm × 10.7 cm). MCA, middle colic artery; AO, aorta abdominalis; IMA, inferior mesenteric artery; RBV, reproductive blood vessels.

The echocardiography in ICU showed moderate systolic dysfunction with the left ventricular ejection fraction of 35%. However, the coronary angiography did not see any sign of myocardial infarction. Furthermore, the thoracic CT showed considerable exudative lesions in both lungs. Meanwhile, abdominal CT (Figures 1A,B) revealed that a vast space-occupying lesion was located in her left middle and upper abdomen and its blood supply mainly came from the inferior mesenteric artery (IMA). The imaging suggested that this may be paraganglioma, but it should differentiate from the mesenchymal malignant tumor such as liposarcoma and fibrosarcoma. Until then, we did not realize the possibility of paraganglioma.

Later, the laboratory test associated with paraganglioma was arranged. The result revealed a signiﬁcant increase in both urine catecholamine and Plasma catecholamine (Table 1, ICU 1st time). Consequently, the diagnosis was considered to be paraganglioma.

**Table 1:** Plasma and urine catecholamine in the timeline.

Parameter	ICU	ICU	Before surgery	After surgery	Pre-discharge	Follow up	Normal value
	(1st time)	(2nd time)				5 months	
Plasma catecholamine							
DA (pmol/L)	78,852	1,0647.6	7,600.9	194.3	<65.2	80.00	≤195.7
E (pmol/L)	16,823	7,245.9	659.9	607.4	137.30	95.00	≤605.4
NE (pmol/L)	93,216	5,702.8	2,354.1	6,302.4	509.60	327.30	414.00–4,435.50
Urine catecholamine							
DA (nmol/24 h)	1,171.17	812	193.87	529.45	55.46	<18.4	4.31–61.60
E (nmol/24 h)	6,214.25	888.17	474.42	1,500.24	109.15	125.92	60.00–352.00
NE (nmol/24 h)	>37,600.00	>72,000.00	>49,600	10,485.49	1,157.52	749.04	750.00–2,088.00

ICU, intensive care unit; DA, dopamine; E, epinephrine; NE, noradrenaline.

With her physical condition markedly improved, she was transferred to our gastrointestinal surgery department for further treatment. However, she was pale, sweaty, and coughed up pink sputum due to constipation the next day. Worse, she felt dyspneic as her degree of blood oxygen saturation decreased gradually. It was considered to be the result of the recurrence of acute left heart failure caused by the catecholamine crisis (Table 1, ICU 2nd time), so she was transferred to ICU again for advanced life support.

Since then, we recognized that the paraganglioma was easily stimulated to release catecholamines which could raise blood pressure and lead to acute heart failure. Imaginably, the next catecholamine crisis may be life-threatening. So, if the condition was permitted, we recommended that the surgery should be performed as early as possible, though the surgery was also a kind of stimulating factor.

After more than 1 month of anti-heart failure therapy and preparation, the surgery was performed under general anesthesia in April 2021. During the operation, we found that peritoneal giant mass with a volume of 17.0 cm × 13.1 cm × 10.7 cm was closely related to the peripheral blood vessels and organs (Figure 1C). Especially, the tumor had a common blood supply to the left half colon (Figures 1C,E). Together with the left hemicolon, the mass was separated carefully and removed entirely from the surrounding tissue (Figure 1F).

The catecholamine in both her urine and plasma returned to normal basically after surgery (Table 1). She developed the lymphatic leak latterly and was discharged from the hospital on the 23rd after surgery with good condition.

**Figure 2:**
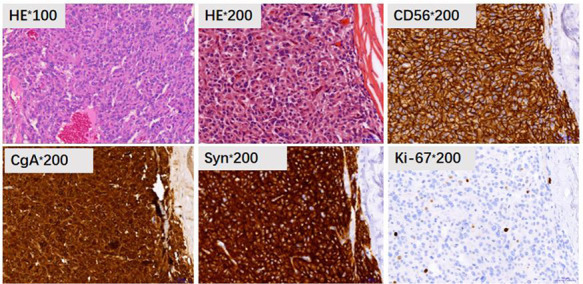
Pathology and immunohistochemistry showed HE staining of the tumor tissue (HE, 100× and 200×), CD56 (+), CgA (+), Syn (+), and Ki-67 index was 15%.

Histological examination showed that the tumor measuring 17.0 cm × 13.1 cm × 10.7 cm, with an intact capsule had localized necrosis. It had invaded the mesentery of the colon as well as para-tumor lymph nodes. Immunohistochemistry showed CD56 (+), CgA (+), Syn (+), CK (−), CD34 (+), SMA (−), CD10 (−), EMA (−), HMB45 (−), Inhibin-α (−), MelanA (−), NF (−), S-100 (−), and the Ki-67 index was 15% (Figure 2). Based on morphology, immunohistochemistry, and clinical presentation, the tumor was finally confirmed as paraganglioma. The patient received genetic testing and no pathogenic genes were found.

## Follow-up

**Figure 3:**
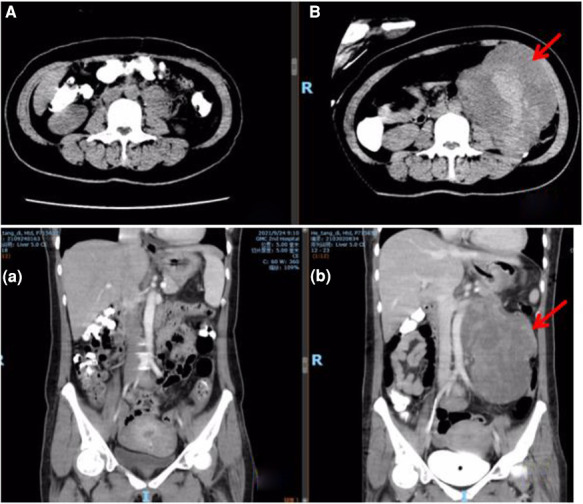
Abdominal CT comparison. (**A/a**) postoperative CT at five months; (**B/b**) preoperative CT (arrow refers to the tumor).

After being followed up for 5 months, the patient was free of abdominal discomfort, sweating, acid reflux, or symptoms of heart failure. Meanwhile, there were no signs of recurrence through CT evaluation (Figure 3).

## Discussion

Paragangliomas are unusual neuroendocrine neoplasms originating from cells derived from the neural crest. This tumor is called pheochromocytoma in the adrenal medulla and is known as extra-adrenal pheochromocytoma elsewhere or simply as paraganglioma (3). The incidence of pheochromocytoma and paraganglioma is about 0.6 cases per 100,000 person-years (4, 5).

Based on the presence or absence of typical clinical signs, retroperitoneal paragangliomas can be classified into three categories: non-functional, sub-clinical, and functional types. Patients with functional paragangliomas that overproduce catecholamines may experience the classic triad symptom including tachycardia, headache, and diaphoresis (6–10). In contrast, those with non-functional retro-peritoneal paragangliomas are often diagnosed incidentally or manifest as compressive symptoms such as abdominal pain that may be associated with nausea, vomiting, abdominal distension, weight loss, or even paralytic ileus (8, 11, 12). Although the sub-clinical type of retro-peritoneal paraganglioma has the function of secreting catecholamines including epinephrine (E), norepinephrine (NE), and dopamine (DA), its secretion is too little to cause typical clinical symptoms and the blood pressure often fluctuates drastically as the stimulating factors trigger the release of catecholamines. This case is a sub-clinical retro-peritoneal paraganglioma. The patient was admitted to our hospital without hypertension, palpitations, diaphoresis, and other catecholamine-associated symptoms. But gastroscopy examination and constipation triggered successive catecholamine crises released from the paraganglioma, which resulted in strong arterial vasoconstriction with a sudden rise in blood pressure as well as coronary spasm that led to myocardial ischaemia and hypoxia. And this mechanism resulted in cardiomyopathy and other complications like acute left heart failure, cardiogenic pulmonary oedema, and pleural effusion. Therefore, it was difficult to diagnose this case due to the atypical clinical symptoms and severe complications.

In differential diagnosis, paraganglioma located in the retro-peritoneum should be differentiated from nerve sheath tumor, liposarcoma, and giant lymph node hyperplasia. Diagnosis is made through laboratory and imaging tests (4). Consequently, metanephrines or their metabolites measurement in both plasma and urine is an excellent diagnostic method. It is recommended as an initial method of diagnosis according to the recommendations of clinical practice of the Society of Endocrinology (5). The ultrasound may be a first-line screening method. CT and MRI have higher sensitivity. Most retroperitoneal paragangliomas are round solid tumors with abundant blood supply, hemorrhagic cystic changes, and calcification. Functional imaging with meta-iodobenzylguanidine (MIBG) scintigraphy may be used for better localization of extra-adrenal disease or metastatic sites (11, 13). Although imaging techniques are helpful, the diagnosis of paragangliomas can only be conﬁrmed with careful histological and immunohistochemical evaluation (14). The combination of immunohistochemical neuronal cell-specific enolase (NSE) and chromogranin (CgA) is up to 100% sensitive.

Paraganglioma with tumor size larger than 5 cm has a metastatic tendency. It is advisable to perform surgical resection to improve survival (15). Resection is often extremely risky since these highly vascular tumors are frequently located near multiple essential blood vessels. Preoperative preparation plays a vital role in the management of paraganglioma. After systematic treatments, the patient's echocardiography result and the manifestation of heart failure were markedly improved. She took phenoxybenzamine and metolol for at least 14 days to normalize blood pressure and heart rate. Treatment also included fluid intake to reverse catecholamine-induced blood volume contraction preoperatively in case of severe hypotension after tumor removal. After that, the resection of the left retroperitoneal tumor was arranged to perform.

During the procedure, we found that the retro-peritoneal mass about 17 cm in diameter was adjacent to the peripheral vessels and organs like the abdominal aorta (AO), the left half colon, the middle colic artery (MCA), the inferior mesenteric artery (IMA), the left genital vessels as well as the left urinary system including the left side of kidney, ureter and renal vessels. Moreover, the tumor had a common blood supply with the left hemicolon (Figure 1C). In case of triggering the catecholamine crisis again by compression roughly, we adopted the open surgical excision through a median cross-cut incision and removed the tumor together with the left half of the colon gently. Of note, we prioritized ligation of tumor venous reflux branches to prevent catecholamines from being released into the blood, followed by the arterial branch of the tumor. In addition, it was essential to keep close collaboration with the anesthesiologist. Meanwhile, monitoring closely of cardiovascular and hemodynamic variables, including intro-arterial pressure and heart rhythm, was also required. Finally, the surgery was completed smoothly without significant complications. Operation time, blood loss, and volume of red blood transfused during the surgery were 210 min, 800 ml, and 6 units, respectively. The patient recovered from the operation after symptomatic treatment and had a good surgical outcome during follow-up. As tumors are said to be unresectable, attempts to reduce their size using chemotherapy, radiation therapy, embolization or molecular-targeted therapy such as tyrosine kinase inhibitor may be indicated (16, 17).

A study showed that the 5-year likelihood of recurrence among those with an extra-adrenal paraganglioma was approximately 20% (18). Hence, patients should be followed up for life. We followed up with the patient 5 months later, and she was disease-free with a normal range of catecholamines in serum and urine, and no imaging evidence of recurrence had been found. It is reported that most cases of metastatic paragangliomas are associated with mutations or hereditary syndromes, such as mutations in rearranged during transfection (RET), neurofibromatosis type 1 (NF1), Von Hippel-Lindau (VHL), and gene mutations of the subunits of succinate dehydrogenase (SDH) (4, 19, 20). Given this condition, our patient had also undergone genetic testing, but the result did not see the above pathogenic genes.

## Conclusion

We presented a rare case of retroperitoneal paraganglioma with successive catecholamine crises as well as acute heart failure. This is probably the largest retro-peritoneal paraganglioma since it was reported in the 1980s (7). Our case emphasizes the inclusion of extra-adrenal paraganglioma in the differential diagnosis of retroperitoneal tumors. In suspected paragangliomas, catecholamine testing is preferable to invasive procedures including gastroscopy and biopsy to avoid triggering a catecholamine crisis. Surgical resection is the mainstay of treatment. Surgical resection is the primary treatment. It is worth mentioning that we are the first to use surgical drawing to illustrate the complex anatomy of huge retroperitoneal paraganglioma. We highlight the priority of dealing with the venous reflux branches of the tumor to prevent the release of catecholamines into the blood. In particular, preoperative preparation plays a vital role in managing paraganglioma. Moreover, it is necessary to schedule genetic testing and clinical follow-up due to the metastatic potential of paragangliomas.

## Data Availability

The raw data supporting the conclusions of this article will be made available by the authors, without undue reservation.
